# Long‐Term Persistence of Hyaluronic Acid Fillers: A Clinical Illustration and Implications for Practice

**DOI:** 10.1111/jocd.70879

**Published:** 2026-04-21

**Authors:** Michael Prager, Joanne Lusher

**Affiliations:** ^1^ The Prager Clinic London UK; ^2^ Regent's University London London UK

## 1 Introduction

Hyaluronic acid (HA) fillers are often widely regarded as temporary and reversible soft‐tissue augmentation agents. However, emerging imaging evidence and long‐term clinical observation suggest that persistent filler material may remain for years following repeated treatments, particularly in patients with measurable cumulative exposure [1]. Early clinical investigations focused primarily on the correction of discrete facial rhytides, most commonly the nasolabial folds. These employed validated grading systems such as the Wrinkle Severity Rating Scale (WSRS) with blinded photographic assessment to quantify improvement and durability [2]. Treatment success was then defined by a measurable reduction in fold severity and time to return toward baseline.

Injection strategies have since evolved toward comprehensive full‐face volumetric rejuvenation. Structural augmentation of the midface, temples, jawline, and chin increasingly supports isolated wrinkle correction [3, 4, 5]. Self‐reported outcome measures have shifted toward global aesthetic improvement scale ratings like the FACE‐Q [6], and while these tools capture overall satisfaction and perceived improvement, they do not quantify cumulative filler burden or long‐term tissue persistence.

Independent observer‐based research demonstrates that nonsurgical facial rejuvenation can measurably alter perceived attractiveness, youthfulness, and health when evaluated by naïve raters [7]. However, systematic third‐party perceptual validation and long‐term imaging follow‐ups have not routinely been incorporated into volumetric filler outcome studies. At the same time, ultrasound and magnetic resonance imaging (MRI) investigations have documented detectable HA filler months to years after injection [8, 9, 10].

We present a case of long‐term HA persistence in a patient with extensive cumulative exposure and discuss potential mechanistic contributors, including volumetric layering and injection technique.

## 2 Clinical Illustration

A middle‐aged female with a long‐standing history of aesthetic treatment presented with concerns regarding progressive facial fullness and focal firmness involving the mid‐face and perioral regions. This patient reported receiving repeated HA filler injections over approximately two decades. Early treatments targeted nasolabial folds. Subsequent sessions expanded to include multi‐regional volumetric augmentation of the cheeks, lips, marionette lines, and jawline. Precise historical treatment records are unavailable. However, based on the duration of treatment, frequency of sessions, and typical volumetric approaches used over this time period, cumulative injected volume was conservatively estimated to exceed 30 mL of HA filler.

The patient's prolonged filler complications resulted in psychological distress and impaired self‐esteem. She expressed repeated fears over worsening disfigurement and concerns relating to how her appearance was perceived by others. This heightened self‐consciousness and preoccupation with self‐image led to feelings of social withdrawal, embarrassment, and shame.

The patient subsequently underwent several sessions of hyaluronidase administration at different time points in attempts to dissolve previously injected filler. Despite these interventions, the patient perceived persistent fullness and textural irregularity. Clinical examination revealed discrete areas of increased subcutaneous density within the malar and perioral regions. These areas were mobile relative to deeper structures and were not associated with erythema, tenderness, or clinical signs of acute inflammation. The overall soft tissue contour appeared fuller than expected for age‐related fat redistribution alone. MRI findings consistent with retained dermal filler material have been publicly disclosed by the patient and are referenced here for contextual support. The imaging demonstrated well‐demarcated foci within the subcutaneous plane corresponding anatomically to prior injection sites. No private medical records were accessed in preparation of this article and the patient provided consent for this publication.

This clinical presentation raises concern for long‐term HA persistence in the setting of repeated cumulative exposure.

## 3 Discussion

Initial HA filler trials were structured around quantifiable correction of specific anatomic defects, particularly nasolabial folds, using validated wrinkle severity grading and blinded evaluator assessment. Durability was measured as the maintenance of fold correction over time. As aesthetic strategies evolved toward whole‐face volumizing treatment, it frequently involved higher cumulative injection volumes across multiple anatomical compartments. Outcome measures became more global, with GAIS and patient‐reported satisfaction scales assessing overall aesthetic improvement rather than discrete line reduction. These instruments are clinically valuable but do not evaluate cumulative filler deposition or long‐term volumetric burden.

Observer‐based attractiveness research has shown that aesthetic interventions can produce measurable changes in external perception of age and attractiveness [7]. Nevertheless, volumetric filler studies rarely incorporate blinded third‐party perceptual evaluation or systematic long‐term imaging follow‐up beyond standard study timelines.

Although HA is biodegradable, imaging literature demonstrates that complete disappearance within traditionally cited timelines is not universal and may be influenced by injected volume and technique. High‐frequency ultrasound and MRI studies have visualized residual filler months and, in some reports, years after treatment [8, 9, 10]. In many individuals, retained material remains clinically silent. However, repeated layering over extended treatment periods may alter local tissue architecture.

Several mechanisms may contribute to this prolonged persistence. High cross‐link stability may promote integration within extracellular matrices. Repeated deposition within identical anatomical compartments can result in cumulative layering. Encapsulation or compartmentalization of larger bolus deposits may reduce uniform enzymatic accessibility. Limited diffusion or insufficient dosing during hyaluronidase administration may further contribute to incomplete degradation [11, 12]. Furthermore, as reviewed by Hong et al., filler migration and granuloma formation represent additional long‐term sequelae that may arise from persistent material and contribute to clinical firmness and contour irregularities [13].

Moreover, needle‐based bolus techniques were widely used in earlier treatment paradigms and frequently deposited discrete filler pools, and larger localized deposits may be less uniformly accessible to enzymatic degradation compared with diffusely distributed microaliquots typically achieved through cannula‐based techniques. This, in patients with extended treatment histories and substantial cumulative exposure, long‐term persistence may therefore represent progressive integration rather than simple delayed biodegradation.

This brief clinical commentary represents a single illustrative case, which may not reflect the outcomes of the wider aesthetic patient population. Precise cumulative injection volumes were unavailable and have been estimated based on treatment duration and typical volumetric approaches. Furthermore, imaging findings were publicly disclosed and were not independently obtained for the purposes of this report. The observations should therefore be interpreted as hypothesis‐generating and underscore the need for prospective and long‐term imaging studies, as this remains an under‐researched area.

These observations do however provide immediate implications for clinical practice, as it remains evident that HA filler persistence beyond expected timelines may occur following repeated cumulative treatments, which can in turn lead to significant psychological distress. Injection technique and volumetric strategy may influence enzymatic accessibility and long‐term tissue integration. Informed consent discussions should therefore include realistic expectations regarding longevity in patients undergoing repeated multiregional augmentation. This point is particularly pertinent for safeguarding against the broader psychosocial impact of long‐term filler complications. Prospective imaging‐based research is warranted to better characterize cumulative volumetric thresholds associated with persistent material.

In conclusion, HA fillers have evolved from region‐specific wrinkle correction tools to instruments of comprehensive volumetric rejuvenation. While generally regarded as temporary and reversible, clinical observation and imaging evidence indicate that long‐term persistence can occur, particularly in the setting of repeated cumulative exposure exceeding typical single‐treatment volumes. As aesthetic paradigms continue to evolve, systematic long‐term evaluation of filler behavior remains crucial to both clinical safety and the protection of patients' psychosocial wellbeing.

## Author Contributions

Both authors contributed to the conception, drafting, and critical revision of the manuscript and approved the final version for submission.

## Funding

No funding was received for this work.

## Ethics Statement

The authors have nothing to report.

## Consent

Informed consent and photo consent were provided by patients.

## Conflicts of Interest

The authors declare no conflicts of interest.

## Data Availability

No new data were generated or analyzed in this correspondence.
